# Genetic associations and phenotypic heterogeneity in the craniosynostotic rabbit

**DOI:** 10.1371/journal.pone.0204086

**Published:** 2018-09-20

**Authors:** James R. Gilbert, Joseph E. Losee, Mark P. Mooney, James J. Cray, Jennifer Gustafson, Michael L. Cunningham, Gregory M. Cooper

**Affiliations:** 1 Department of Plastic Surgery, University of Pittsburgh/Children’s Hospital of Pittsburgh, Pittsburgh, Pennsylvania, United States of America; 2 McGowan Institute of Regenerative Medicine, Pittsburgh, Pennsylvania, United States of America; 3 Department of Oral Biology, University of Pittsburgh, Pittsburgh, Pennsylvania, United States of America; 4 Department of Anthropology, University of Pittsburgh, Pittsburgh, Pennsylvania, United States of America; 5 Department of Orthodontics, University of Pittsburgh, Pittsburgh, Pennsylvania, United States of America; 6 Oral Health Sciences, Medical University of South Carolina, Charleston, South Carolina, United States of America; 7 Center for Developmental Biology and Regenerative Medicine and the Craniofacial Center Seattle Children’s Hospital, Seattle, Washington, United States of America; 8 Department of Bioengineering, University of Pittsburgh, Pittsburgh, Pennsylvania, United States of America; National Cheng Kung University, TAIWAN

## Abstract

Craniosynostosis (CS) is a disorder that involves the premature ossification of one or more cranial sutures. Our research team has described a naturally occurring rabbit model of CS with a variable phenotype and unknown etiology. Restriction-site associated DNA (RAD) sequencing is a genomic sampling method for identifying genetic variants in species with little or no existing sequence data. RAD sequencing data was analyzed using a mixed linear model to identify single nucleotide polymorphisms (SNPs) associated with disease occurrence and onset in the rabbit model of CS. SNPs achieving a genome-wide significance of p ≤ 5 x 10^−8^ were identified on chromosome 2 in association with disease occurrence and on chromosomes 14 and 19 in association with disease onset. Genotyping identified a coding variant in fibroblast growth factor binding protein 1 (*FGFBP-1*) on chromosome 2 and a non-coding variant upstream of integrin alpha 3 (*ITGA3*) on chromosome 19 that associated with disease occurrence and onset, respectively. Retrospective analysis of patient data revealed a significant inverse correlation between *FGFBP-1* and *ITGA*3 transcript levels in patients with coronal CS. *FGFBP-1* and *ITGA3* are genes with roles in early development that warrant functional study to further understand suture biology.

## Introduction

During normal development the cranial sutures consist of a fibrous and highly cellular connective tissue that bridges the bones of the cranial vault. These joints function as growth sites until the brain reaches its mature volume. Craniosynostosis may result in restricted cranial growth, altered intracranial volume, elevated intracranial pressure, and developmental impairment of other vital structures [1, 2]. The clinical presentation of CS is complex and may vary in terms of age-of-onset and suture involvement. Mutations in more than 50 genes recurrently cause craniosynostosis (reviewed by Twigg and Wilkie, 2015) [3]. The fibroblast growth factor signaling pathway was among the first molecular mechanisms linked to CS and errors in FGF signaling remain the most common cause of CS in cases with a defined etiology. Other mutations associated with CS affect receptor kinase signaling, developmental transcription factors, and cell-cell or cell-matrix interactions. Pathway analysis of gene expression data has implicated extracellular matrix (ECM) interactions as differentially regulated gene networks in cases of single-suture synostosis [4]. Despite this progress, the molecular basis for disease remains uncertain in approximately 80% of patients affected by CS.

Mooney and colleagues first reported a colony of rabbits with naturally occurring, familial synostosis of the coronal suture in 1993 [5–7]. The colony remains the only reproducible large animal model of CS in existence. Published work describes the initial identification, phenotypic variability, and breeding demographics in rabbits from this colony [5–8]. Disease progression in the CS rabbit ranges from early-onset (EOS) to postnatal delayed-onset (DOS) with unilateral or bilateral fusion of the coronal suture [9–11]. Recent work suggests a modifying allele may determine the age-of-onset for coronal suture fusion [9], though the genetic etiology remains undefined. The current study was designed to identify the loci responsible for occurrence and the timing of suture fusion within the synostotic rabbit.

## Materials and methods

### Animal housing

All research animals were maintained and bred within the Animal Facility Core located within the Rangos Research Building of the University of Pittsburgh/Children’s Hospital of UPMC of Pittsburgh. Special dispensation was allowed for individual caging of breeding animals due to the combative nature of male rabbits and breeding susceptibility of female rabbits. All other housing and husbandry was in accord with The Guide for the Care and Use of Laboratory Animals and The Animal Welfare Act. Potentially pregnant females were provided a nutritionally supplemented breeder diet as recommended by veterinary staff. Pregnant females were provided extra housing space and breeder boxes for nesting/delivery. Infant rabbits were housed with the mother up to 42 days post-delivery.

### Animal model

The craniosynostotic rabbit colony maintained at the University of Pittsburgh has been described [5–7, 9, 12]. One hundred four rabbits were used in this study. Diagnosis of colony rabbits was based on previously published criteria. Animals exhibiting coronal suture fusion at 10 days of age were diagnosed as EOS. In animals that exhibited coronal suture mobility at day 10, holes were made in the periosteum and bone using a fine dental burr (0.4 mm) and packed with silver dental amalgam to serve as radiopaque markers. The holes were placed in quadrants, 2 mm anterior and posterior to the coronal sutures, and 2 mm lateral to the sagittal and interfrontal sutures. Cephalographs of the rabbits were taken at 10 and 25 days of age and rabbits in which coronal suture marker separation fell below the 95% confidence interval of the mean for unaffected rabbits were diagnosed as DOS. Rabbits with DOS synostosis average about 70–75% of normal coronal suture growth and usually fuse by 42 days of age [6, 13–16]. All other colony rabbits were diagnosed as ICN.

### Tissue samples

Ear snips (~50 mg/animal) were collected at the time of diagnosis or blood samples (~1 mL) were drawn from the marginal ear vein in older rabbits during routine veterinary care. Additional tissues were harvested after euthanasia during routine culling of the breeding colony. This study was reviewed and approved by the University of Pittsburgh, Institutional Animal Care and Use Committee (IACUC).

### RAD-sequencing and variant calling

Genomic samples were submitted to Floragenex (Portland, OR) for RAD-Sequencing. Samples were digested with Pst I and were multiplexed with unique barcodes prior to sequencing. A total of 183.4 million reads (mean value = 3,743,339 reads/animal) were obtained from the submitted samples. Sequence alignment files and study metadata have been made available through the European Nucleotide Archive under study accession number PRJEB26920 using sample accession numbers ERR2595558-ERR2595603. Variant calling was performed with the minimum Phred score for genotyping set to 15, the minimum sequence coverage for genotyping set to 4, and a minimum of 65% of the population genotyped. A total of 85,908 variant loci were identified using these parameters and 53,406 of these loci could be mapped to autosomal positions in the OryCun2.0 reference assembly. The variant call file and metadata have been made available through the European Variation Archive under accession number PRJEB27278.

### Association testing

Genetic associations with disease phenotype were analyzed using an identity-by-state kinship matrix and an Efficient Mixed-Model Association eXpedited (EMMAX) algorithm as implemented in Sequence Variation Suite software (Golden Helix, Bozeman, MO) [17]. SNPs were excluded from analysis if calls were missing in more than 10% of the samples or if the MAF = 0.0. All synostotic animals (N = 24) were compared to ICN controls (N = 22) in order to identify SNPs associated with disease occurrence. EOS synostotic animals (N = 12) were compared to DOS controls (N = 12) in order to identify SNPs associated with age-of-onset.

### Linkage analysis

Linkage disequilibrium (LD) was estimated using the Haploview software package and the confidence interval (CI) method of Gabriel *et al* (upper CI = 0.95, lower CI = 0.55, fraction of strong LD in informative comparisons ≥ 0.85) [18]. In order to evaluate LD associated with disease occurrence coarse mapping of 128 SNPs ranging from position 2,001,654 to position 9,644,295 on chromosome 2 was performed using case-control populations of affected (N = 24) and unaffected (N = 22) control animals. A single SNP with genome-wide significance (p ≤ 5 x 10^−8^) was observed to occur within a linkage block that ranged from position 6,388,292 to position 6,641,309 on chromosome 2. Targeted mapping of this region was subsequently performed using 12 tagged SNPs defining the interval. In order to evaluate LD associated with disease onset coarse mapping of 76 SNPs ranging from position 37,074,897 to position 38,090,500 on chromosome 19 was performed using case-control populations of EOS CS rabbits (N = 12) and DOS CS rabbits as controls (N = 12). A single linkage block was identified ranging from position 37,533,454 to position 37,824,932 on chromosome 19. Targeted mapping of this region was subsequently performed using 6 tagged SNPs defining the interval.

### Candidate gene prioritization

Candidate genes were ranked based upon association with signaling pathways involved in craniosynostosis, phenotypes listed in the Human Gene Mutation Database, and/or by scoring with Phenolyzer software [19, 20]. Key terms used for scoring included “bone,” “cartilage,” “musculoskeletal,” “craniofacial abnormalities,” and “craniosynostosis.”

### Sequencing

*FGFBP-1* (ENSOCUG00000016098) and the *ITGA3* upstream region (ENSOCUT00000009992.3) were amplified from the rabbit genome using the following primer pairs:

FGFBP-1 Forward 5’-ATGAGGATCCAAAGTCTCACCCTGCTCTCCATCCTCCTCCTGGC-3’
FGFBP-1 Reverse 5’-TCAGCACGACCTGTCCTGTATCATGGTGAGGAAGAATGTGC-3’
ITGA3 Forward 5’-GCCACCTCCTGGGCACTCACAGCGC-3’
ITGA3 Reverse 5’-GCCTCTCCCGCAGTCGGGTCACCGGC-3’.

Purified PCR products were submitted to GENEWIZ (South Plainfield, NJ) for sequencing. Sequencing alignments were performed using Clustal Omega (EMBL-EBI, Cambridge, UK). Chromatograms were visually inspected using Chromas v.2.5.1 Software (Technelysium, South Brisbane, Australia) for validation of sequencing results.

### Bioinformatic analysis of predicted amino acid substitutions

The potential impact of amino acid substitutions at amino acid 158 (*Thr*158*Ile*) and amino acid 175 (*Gln*175*Arg*) of the FGFBP-1 protein was evaluated using the Polyphen 2.0 software package (Bork Group & Sunyaev Lab, Harvard, web software, http://genetics.bwh.harvard.edu/pph). ClustalW (http://www.ebi.ac.uk/Tools/msa/clustalw2/) alignment of the FGFBP-1 protein sequence for conservation analysis was performed using twelve species including: *Cercocebus atys* (XP_011900804.1), *Chlorocebus sabaeus* (XP_008016034.1), *Gorilla gorilla* (XP_004038517.1), *Homo sapiens* (NP_005121.1), *Macaca fascicularis* (XP_005554572.1), *Mandrillus leucophaeus* (XP_011839494.1), *Microcebus murinus* (XP_012593005.1), *Nomascus leucogenys* (XP_003258554.1), *Pan troglodytes* (XP_009445659.1), *Pongo abelii* (XP_002832895.1), *Rhinopithecus bieti* (XP_017730851), and *Rhinopithecus roxellana* (XP_010356426.1).

### Cell harvest and RNA isolation from human patient samples

RNA was isolated from human patient samples as previously described [4]. For RNA extraction, Roche High Pure miRNA Isolation Kit was used with accordance to the manufacturer's protocol (Roche). RNA was stored at −80°C and submitted for microarray processing on dry ice.

### Microarray analysis of human patient samples

RNA integrity was assessed using the Agilent 2100 Bioanalyzer, and only samples passing quality control were analyzed for transcriptomic changes using Affymetrix Human Gene 1.0 ST arrays, on which 28,869 genes are represented. Raw microarray data was processed and analyzed with Bioconductor. Microarray quality control metrics include the manufacturer's recommended guidelines: (1) visual inspection of probe array images, (2) proper ranking of hybridization and Poly-A controls, and (3) area under the curve values for a receiver operating characteristic plot comparing the positive control and negative control signal values. Other microarray quality control metrics from the Bioconductor affyPLM package were used, including the relative log expression (RLE) values, used to see if expression values are shifted or spread out, and the normalized unscaled standard errors (NUSE), used to see if the variability of genes across arrays is too large. To identify a set of genes whose expression levels vary significantly across the population, singular value decomposition (SVD) of the normalized data for each probe set was performed and the percent variance explained by the 1st singular value was investigated. This value is referred to as the Gene Information Content (GIC). A cutoff for significant GIC scores was defined by permuting the probe-to-probe set map and calculating the percent variance explained for each permuted probe set. This was repeated one thousand times and the cutoff was defined as the 99th percentile of the permuted statistics. Probes whose observed GIC was less than this value was removed from downstream analyses. All microarray data are MIAME compliant and the raw dataset has been deposited in the MIAME compliant Gene Expression Omnibus (GEO) database under accession number GSE27976 (http://www-ncbi-nlm-nih-gov.offcampus.lib.washington.edu/geo/).

### Statistical methods

Power analysis was performed for case-control study of discrete traits using the Genetic Power Calculator [21]. A total of 24 cases would have 80% power for testing with two degrees of freedom given a risk allele frequency of 0.5, a relative risk of 5.0, *D*’ = 0.85, and α = 0.05. Association tests and were carried out using a single-locus mixed linear model and Manhattan plots were generated using Sequence Variation Suite software (Golden Helix, Bozeman, MO). LD plots were obtained using HaploView v4.06 [22]. Logistic regression was performed using the Real Statistics Resource Pack software [23]. Linear regression was performed using SPSS software (IBM, Armonk, NY).

## Results and discussion

**Fig 1:**
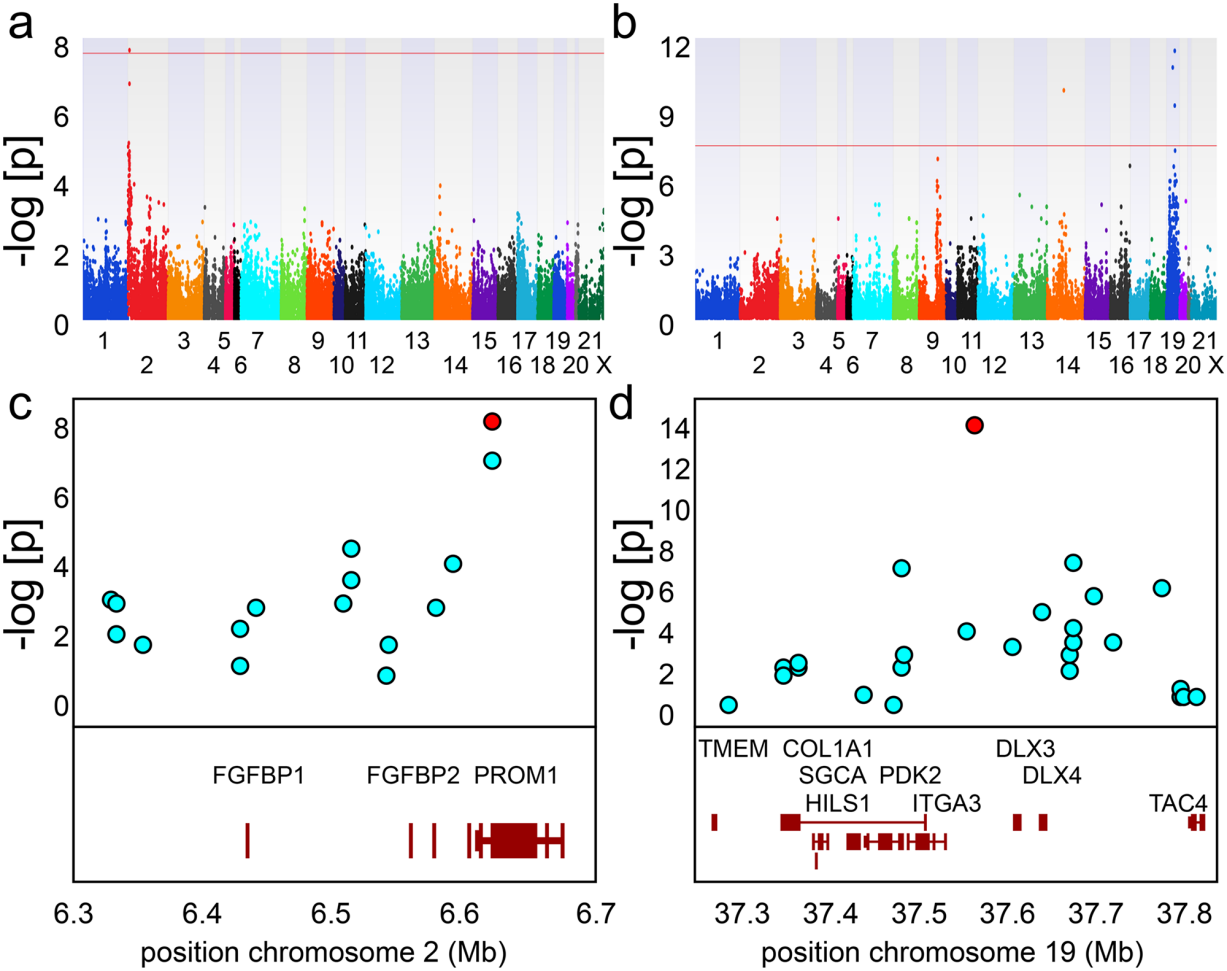
Graphical representation of p values obtained from MLM analysis of RAD sequencing data. Panels correspond to disease occurrence (1a, 1c) and age-of-onset (1b, 1d). For Manhattan plots (1a, 1b) the x axis corresponds to the genomic position of the autosomes and the y axis shows the −log10 of the p value. The horizontal red line corresponds to the genome-wide significance threshold of p ≤ 5 × 10^−8^. For regional association plots (1b, 1d) the x axis corresponds to the chromosomal position and the y axis shows the −log10 of the p value. Genes in the region are shown below (not to scale).

**Fig 2:**
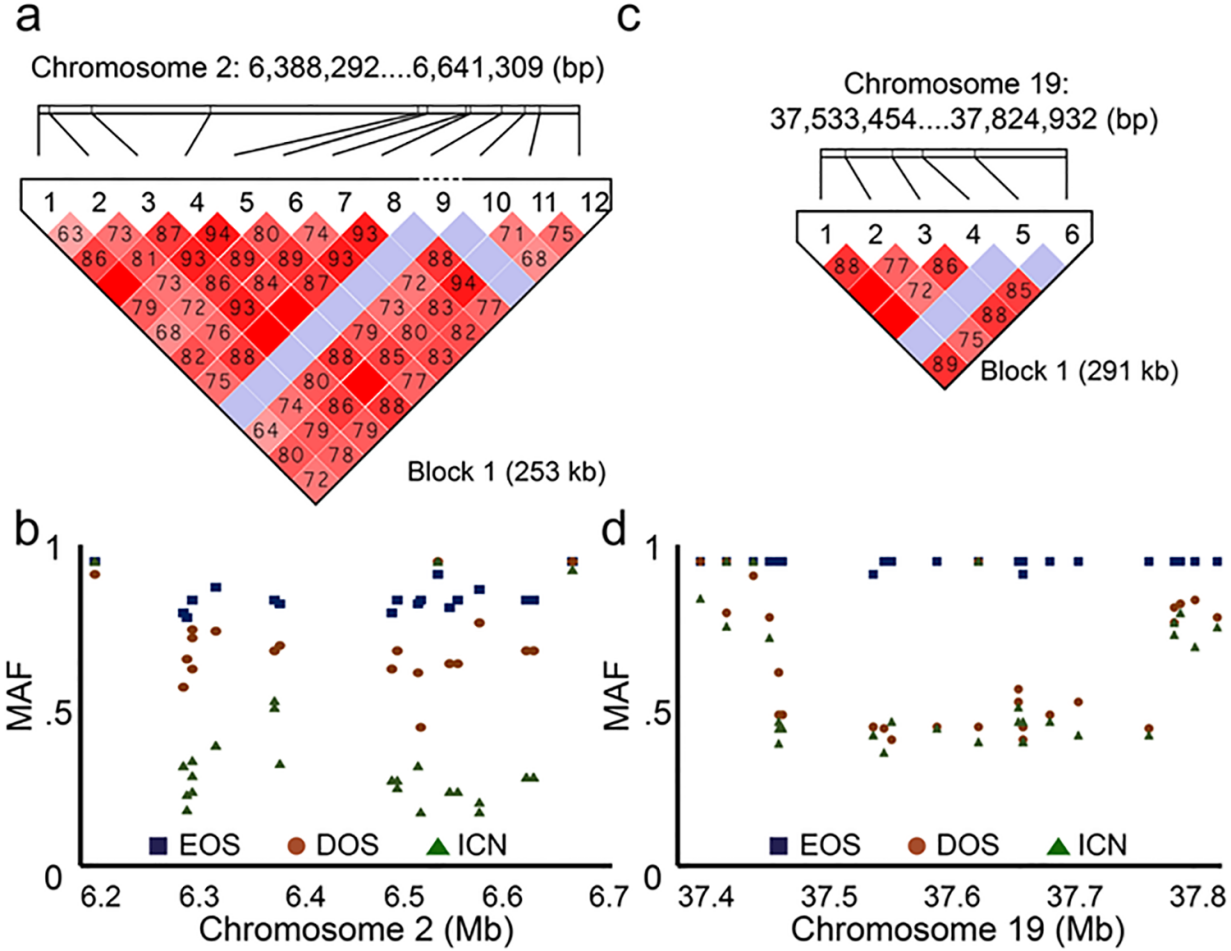
Linkage disequilibrium pattern (D’) and minor allele frequencies (MAF) for disease occurrence (2a, 2b) and age-of-onset (2c, 2d). Linkage blocks were identified in Haploview (2a, 2c). A standard color scheme is used to display LD with bright red color for very strong LD (LOD = 2 D' = 1), white color for no LD (LOD<2, D'<1), pink red (LOD = 2 D'<1), and blue (LOD<2 D' = 1) for intermediate LD. Analysis of Variance (ANOVA) revealed significant differences in allelic distribution for all groups within the linkage block on chromosome 2 and between EOS rabbits and all other populations within the linkage block on chromosome 19.

To identify susceptibility loci associated with disease phenotype in the CS rabbit genomic DNAs obtained from EOS CS rabbits (N = 12), DOS CS rabbits (N = 12), and in-colony normal rabbits (ICN; N = 22) were subjected to RAD sequencing. Diagnosis was performed as previously described by our research group (S1 Fig) [6, 7, 9–12, 24–26]. After stringent quality filtering, 85,908 SNPs were identified. This yield is comparable to the values obtained using RAD sequencing in other vertebrate and plant species including geese, rainbow trout, soybean, and barley [27–30]. Genetic associations were analyzed using a mixed linear model as implemented in Sequence Variation Suite software (Golden Helix, Bozeman, MO). In order to identify loci associated with disease occurrence, the synostotic population was analyzed against ICN control animals. A single SNP reaching the genome-wide significance threshold of p ≤ 5 × 10^−8^ was identified at position 6,641,349 on chromosome 2 (Fig 1a and Table 1). In order to identify loci associated with the age-of-onset within the synostotic population, EOS CS rabbits were analyzed using DOS CS rabbits as the control population. Five SNPs, 1 on chromosome 14 and 4 on chromosome 19, were significantly associated with disease onset (Fig 1b and Table 1). The SNP on chromosome 2 occurred within a 253 kb block of linkage disequilibrium (LD) spanning the coding regions for three genes including *FGFBP-1* (2:6,429,887–6,430,630), *FGFBP-2* (2:6,565,566–6,566,246), and *PROM1* (2:6,570,949–6,622,803) (Fig 1c). We evaluated LD in our samples with an average interval of 21 kb between tagged SNPs (Fig 2a). Heterozygosity values for the SNPs varied from 0.022 to 0.5 within the population, averaging to 0.31 within the synostotic population and 0.39 within the ICN control population (S1 Table). Hardy-Weinberg p values ranged from 0.0098 to 0.6 with a total of four SNPs achieving significance (p ≤ 0.05). Minor allele frequency (MAF) mapping was performed for all SNPs within the defined linkage block (Fig 2b). Analysis of Variance (ANOVA) revealed significant differences in allelic distribution for all groups within the linkage block on chromosome 2 with mean values of 0.878 ± 0.006 for EOS rabbits, 0.72 ± 0.015 for DOS rabbits, and 0.37 ± 0.028 for ICN rabbits (F = 92; p ≤ 0.00001). Of the five SNPs associated with disease onset, three occurred on chromosome 19 at positions 37,528,209 (p ≤ 5.41 x 10^−13^), 37,090,046 (p ≤ 1.81 x 10^−10^), and 38,090,582 (p ≤ 2.04 x 10^−8^) (Fig 1d). Coarse mapping of this region on chromosome 19 identified a 291 kb block of disequilibrium spanning nine genes (Fig 2c and S2 Table). The average interval between tagged SNPs within this linkage block was 48.5 kb. Heterozygosity values for the SNPs within this linkage block varied from 0.048 to 0.417, averaging to 0 within the EOS population and 0.57 in the DOS population (S1 Table). Hardy-Weinberg p values ranged from 0.1463 to 1 and failed to achieve significance. Minor allele frequency (MAF) mapping was performed for all SNPs within the defined linkage block on chromosome 19 (Fig 2d). Significant differences in allelic distribution were observed between EOS rabbits and all other populations by ANOVA with means of 0.95 ± 0.003 for EOS rabbits, 0.65 ± 0.039 for DOS rabbits, and 0.61 ± 0.04 for ICN rabbits (F = 85; p ≤ 0.00001). The two remaining SNPs that associated with disease onset occurred on chromosome 14 at position 72,174,768 (p ≤ 3.67 x 10^−11^) and on chromosome 19 at position 27,723,389 (p ≤ 3.18 x 10^−12^). The only known genes located within 250 kb of either SNP included *TBL1XR1* on chromosome 14 and *TBX2* and *TBX4* on chromosome 19.

**Table 1:** SNPs associated with disease occurrence and age-of-onset in the CS rabbit.

MODEL	CHR	POS	P	FDR	VAR	MAF	X^2^	RR
Fusion	2	6,641,349	1.01 x 10^−08^	4.0 x 10^−4^	0.53	0.49	39	.175
Onset	19 19 14 19 19	37,528,209 27,723,389 72,174,768 37,090,046 38,090,582	5.41 x 10^−13^ 3.18 x 10^−12^ 3.67 x 10^−11^ 1.81 x 10^−10^ 2.04 x 10^−8^	1.19 x 10^−9^ 6.64 x 10^−9^ 7.79 x 10^−8^ 3.42 x 10^−7^ 3.68 x 10^−5^	0.91 0.89 0.87 0.85 0.77	0.26 0.26 0.30 0.30 0.27	17 17 22 18 20	.46 .46 .36 .42 .4

SNPs with genome-wide significance. Comparison of RAD sequencing data from synostotic rabbits (N = 24) with normal controls (N = 22) identified a SNP on chromosome 2. Comparison of EOS CS rabbits (N = 12) with DOS CS rabbits (N = 12) identified 4 SNPs on chromosome 19 and 1 SNP on chromosome 14. CHR = chromosome, POS = position, FDR = false discovery rate, VAR = proportion of variability explained, MAF = minor allele frequency, X^2^ = chi square, RR = relative risk.

Of the three candidate genes identified on chromosome 2, *FGFBP-1* and *FGFBP2* were prioritized for further investigation given the established role of FGF signaling in CS and based on scoring of known phenotypes in the Human Gene Mutation Database (HGMD) [19]. Exonic sequencing of *FGFBP-1* and *FGFBP-2* identified an A→G transition at chromosomal position 2:6,430,107 that was found to have a significant association with disease occurrence (N = 115; X^2^ = 64; p ≤ 4.7 x 10^−18^). This variant resulted in a *Gln*→*Arg* amino acid change at amino acid position 175 of the FGFBP-1 protein and was moderately damaging based on bioinformatic protein prediction tools and conservation analysis. Other variants identified within the colony failed to achieve genome-wide significance and were benign. In order to prioritize candidate genes potentially associated with age-of-onset in the CS rabbit, all candidates on chromosomes 14 and 19 were analyzed using Phenolyzer software (S2 Fig) [20]. The genes *ITGA3* and *COL1A1* scored most highly in Phenolyzer and were prioritized for further investigation. Sequencing of the *ITGA3* cDNA and 5’ upstream region identified a single C→G transversion located at chromosomal position 19:37,599,083 that achieved genome-wide significance (N = 83; p ≤ 1.8 x 10^−11^) and 13 intragenic variants that failed to achieve genome-wide significance. The C→G transversion at chromosomal position 19:37,599,083 occurred within a putative *Ets-1* transcription factor binding site located 573 base pairs upstream of the *ITGA3* transcription start site. Sequencing results for *COL1A1* have previously been reported by our laboratory [31].

**Fig 3:**
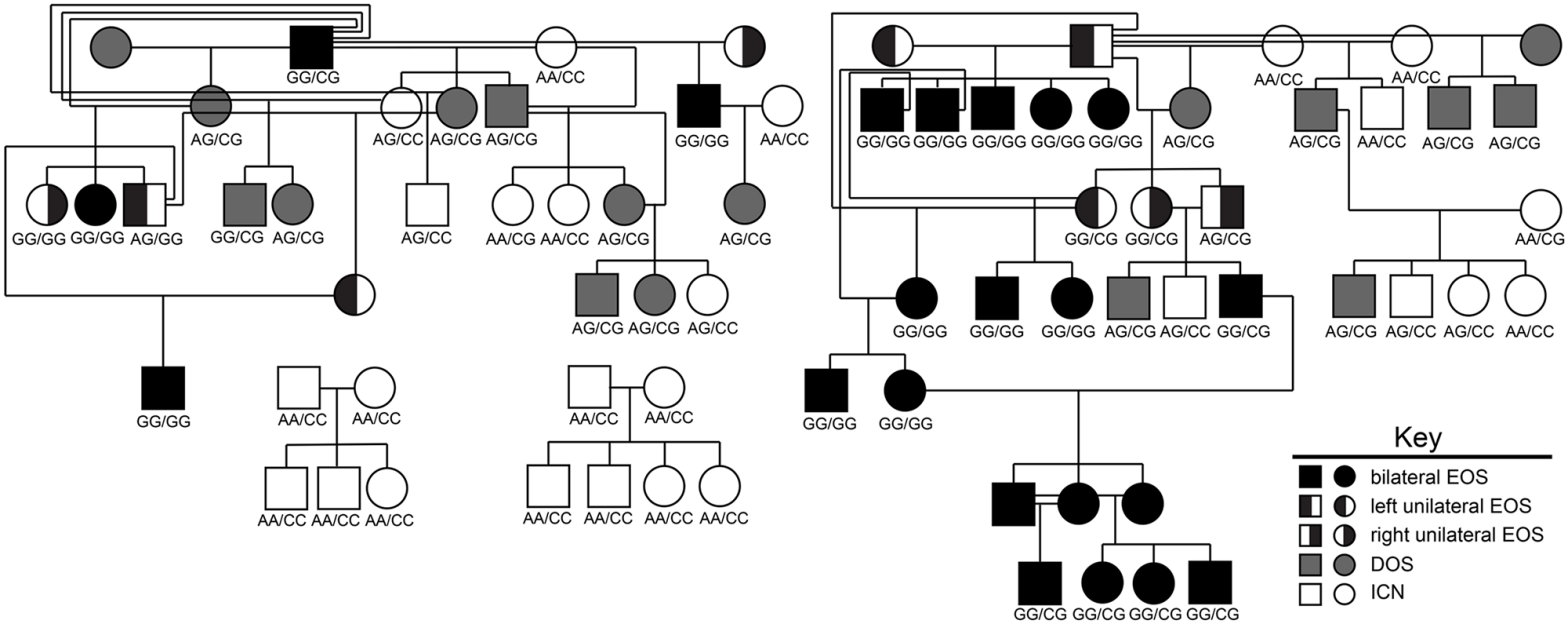
Pedigree of the CS rabbit colony. Colony animals were retrospectively genotyped for an A → G transition located within the *FGFBP-1* coding region at chromosomal position 2:6,430,107 and for a C → G transversion located upstream of *ITGA3* at chromosomal position 19:37,599,083. Biallelic genotypes are reported for *FGFBP-1*/*ITGA3*. Color-coding for phenotype is summarized in the figure key.

**Fig 4:**
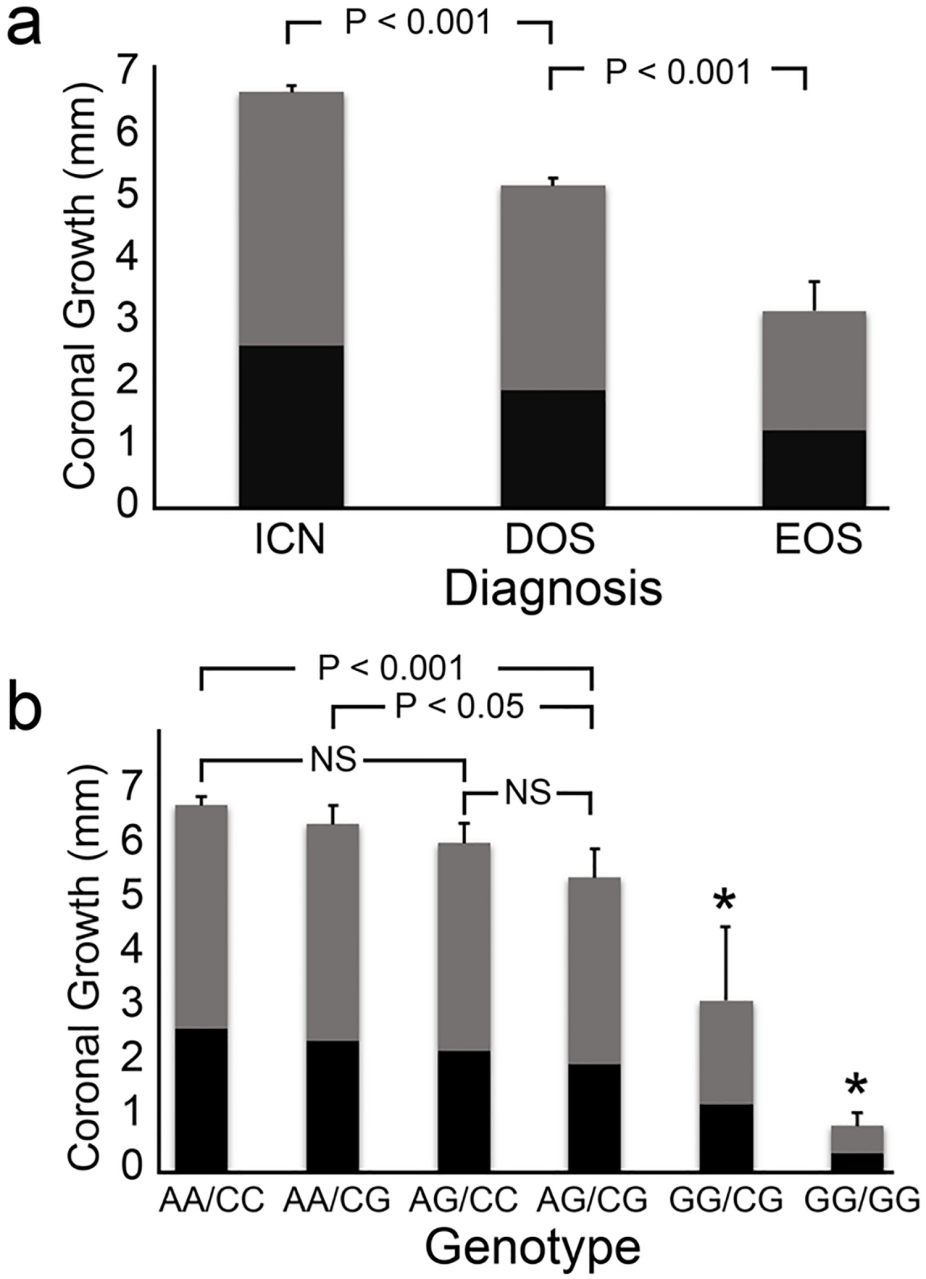
Growth across the coronal suture by diagnosis and by genotype. Radio-opaque amalgam markers anterior and posterior to the coronal suture during initial diagnosis at 10 days of age. Radiographic growth measurements were made at day 25 (■) and day 42 (■) as described in Methods. Two-way ANOVA revealed significant differences in growth rates when animals were grouped by diagnosis (4a). More granular distinctions were evident within the population when animals were grouped by genotype (4b). Progressive accumulation of variant alleles at the two loci correlated with increasing growth restriction. Significant growth restriction was evident in heterozygotes with the AG/CG genotype when compared to the reference AA/CC genotype (p < 0.001) or to the single variant AA/CG genotype (p < 0.05). Animals with the GG/CG genotype exhibited significantly different growth compared to all other groups (p ≤ 0.0001) and animals with the GG/GG genotype exhibited significantly different growth compared to all other groups (p ≤ 0.0001) as denoted by an asterisk (*).

We considered that epistatic interactions might explain the phenotypic diversity observed in the CS rabbit colony. Two-way gene-gene interactions between significant SNPs within the discovery set and between significant SNPs identified by candidate gene sequencing were evaluated using a logistic regression model (Table 2). All interactions were significant with the most significant interaction occurring between the variants identified within *FGFBP-1* and upstream of *ITGA3* (X^2^ = 97, p ≤ 4.8 x 10^−20^). Retrospective analysis of the *FGFBP-1*/*ITGA3* genotype within a pedigree spanning seven generations suggests a digenic mode of inheritance that requires at least one copy of each variant allele for the synostotic phenotype to be expressed (Fig 3). Correlations between animal genotype, phenotypic diagnosis, and growth across the coronal suture were analyzed by two-way Analysis of Variance (ANOVA). Significant group and time main effects and a significant group x time interaction were associated with coronal growth when groups were defined either by diagnosis or by genotype (Fig 4). Post-hoc analysis using Tukey’s HSD for multiple comparisons indicated that the growth rate for animals with the reference AA/CC genotype was significantly different than for animals with the AG/CG, GG/CG, or GG/GG genotypes (p ≤ 0.001). Animals with the AA/CC, AA/CG, and AG/CC genotypes were not significantly different. Heterozygotes with the AG/CG genotype were significantly different from all other groups except those with the AG/CC genotype (p < 0.05). The GG/CG and GG/GG genotypes were each associated with significant differences in growth relative to each other and to all other groups (p < 0.0001).

**Table 2:** Epistatic interactions between SNPs associated with disease occurrence and age-of-onset in the CS rabbit.

SNP Source	Locus 1		Locus 2		Logistic regression		
Sequencing	CHR	POS	CHR	POS	LL	X^2^	epistatic P value
RAD-Seq	2 2 2 2 2	6,641,349 6,641,349 6,641,349 6,641,349 6,641,349	14 19 19 19 19	72,174,768 27,723,389 37,090,046 37,528,209 38,090,582	2.9 2.9 2.8 2.5 2.8	52.2 52.8 57.3 55.9 56.3	1.25 X 10^−10^ 9.13 x 10^−11^ 1.04 x 10^−11^ 2.1 x 10^−11^ 1.72 x 10^−11^
Candidate	2	6,430,107	19	37,599,083	15.5	97	4.8 x 10^−20^

Epistatic interactions evaluated by logistic regression. Epistatic interactions between SNPs associated with disease occurrence and age-of-onset were evaluated using a logistic regression model. Paired loci were drawn from the discovery SNP set and from variants identified by candidate gene sequencing.

**Fig 5:**
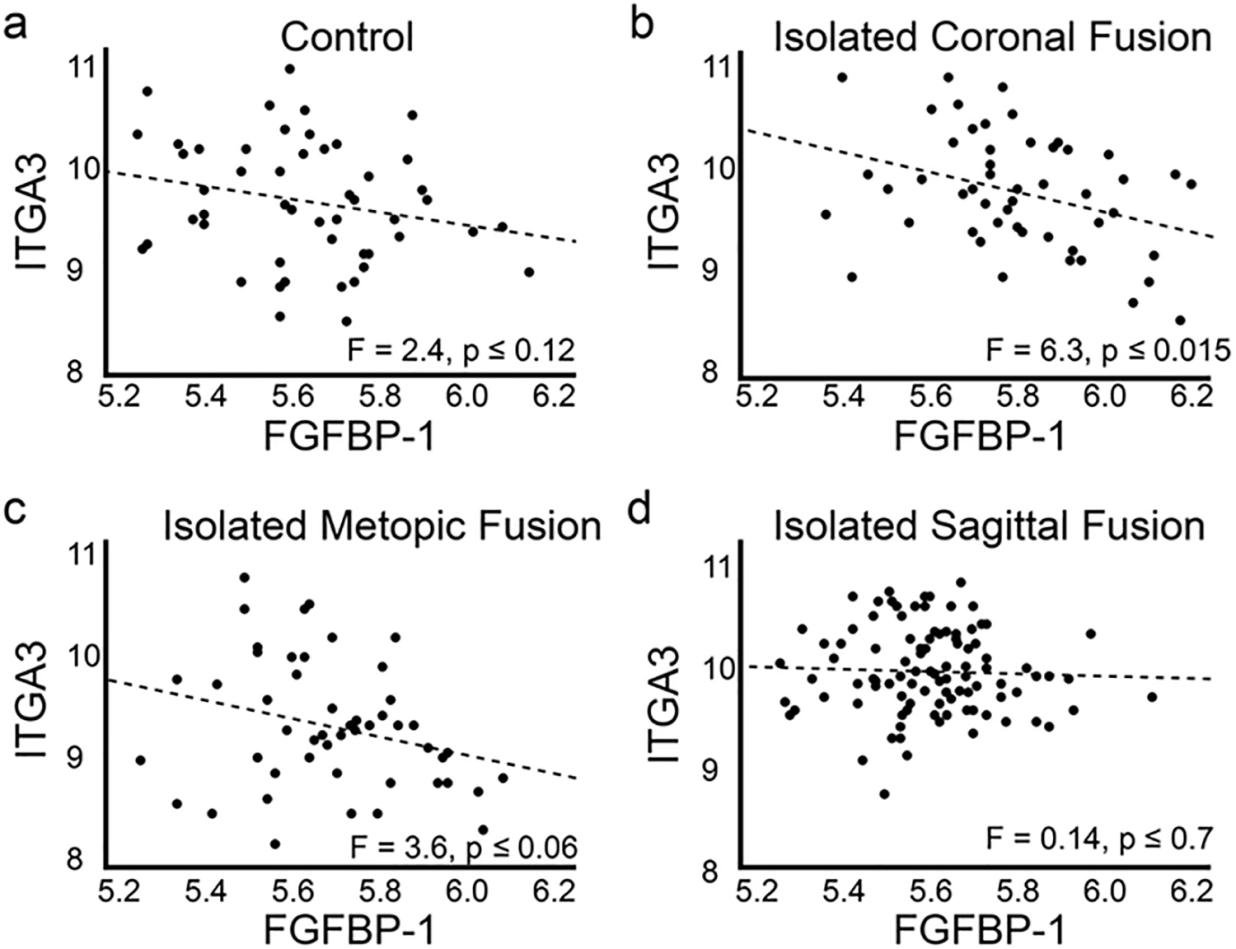
*FGFBP-1* expression negatively correlates with *ITGA3* expression in patients with fusion of the coronal suture but not in patients with fusion of the metopic or sagittal sutures. Scatter plots describing the correlation between *FGFBP-1* and *ITGA3* expression were prepared for a patient cohort consisting of 199 patients and controls [4]. Patient samples were associated with isolated fusion of the coronal, metopic, or sagittal sutures. No correlation was evident within the control population (5a). A significant (p ≤ 0.0155) negative correlation was evident in patients with isolated coronal suture fusion (5b). Correlation of gene expression with isolated metopic (5c) or sagittal (5d) suture fusion fell below the level of significance suggesting that this interaction may be unique to the coronal suture.

We also asked whether *FGFBP-1* and *ITGA3* gene expression might correlate with the synostotic phenotype in humans. To address this question we made use of a previously described transcriptomic data set consisting of clinical samples obtained from unaffected control patients (N = 50), patients with isolated fusion of the coronal suture (N = 50), patients with isolated fusion of the metopic suture (N = 49), and patients with isolated fusion of the sagittal suture (N = 100) [4]. Group-based comparisons of gene expression were initially performed by ANOVA. No significant differences in the expression of *FGFBP-1* were detected between groups. Significant differences in the expression of *ITGA3* were detected between groups (F = 7.3; p < 0.0001). Post-hoc analysis using Tukey’s HSD for multiple comparisons indicated that *ITGA3* expression was reduced in patients with isolated fusion of the coronal suture relative to unaffected control patients (p = 0.025) and patients with isolated fusion of the sagittal suture (p < 0.0001). In light of the epistatic interactions observed in the CS rabbit, we also asked whether expression of *FGFBP-1* and *ITGA3* might correlate within patient populations. Linear regression analysis plotting *FGFBP-1* expression against *ITGA3* expression identified a negative correlation in patients with isolated fusion of the coronal suture (N = 50; p ≤ 0.015) but not in controls (N = 50; p ≤ 0.12) or in patients with isolated fusion of the metopic (N = 49; p ≤ 0.06) or sagittal (N = 100; p ≤ 0.7) sutures (Fig 5). That this correlation is unique to patients with isolated coronal suture fusion is notable given that the rabbit model is characterized specifically by fusion of the coronal suture, although it remains unclear whether this correlation plays any role in the pathology of coronal suture fusion. Further investigation will be required to address the question of epistatic interactions in the pathology of CS.

The first example of digenic inheritance in patients with CS, involving BMP2 and SMAD6 alleles, has recently been described [32, 33]. The work presented in the current study provides the first evidence supporting a digenic mode of inheritance in an animal model of CS. Moreover, the candidates identified within this study provide potential insight into the pathology of CS although it should be noted that higher resolution mapping might have led to the identification of additional candidates. The identification of *FGFBP-1* as a candidate gene in the etiology of CS highlights a previously unidentified avenue through which growth factor stimulation may affect craniofacial development and/or dysmorphology. Whereas FGF receptor mutations linked to CS result in chronic activation of the signaling pathway, FGFBP-1 is an ECM-bound protein that presents matrix-bound FGF ligand to its cognate receptor. Errors in ligand presentation may easily be predicted to alter growth factor signaling kinetics.

We identified two biologically plausible candidate genes in our study, *FGFBP-1* and *ITGA3*, both of which are involved in skeletal development. Patterns of allelic segregation suggest a complex pattern of inheritance and suggest a phenotypic spectrum that correlates with the accumulation of damaging alleles. The role of FGF signaling and evidence of reduced *ITGA3* expression in patients with CS are well established within the literature [4, 34, 35]. Functional investigation is required to define the role of these candidates in craniofacial development and pathology but falls beyond the scope of the present study. The contribution of these loci to craniofacial development is worthy of investigation. Functional studies are required to define the role of these candidates in craniofacial development and pathology. Our results suggest new avenues for investigating the role of epistasis and ECM-interactions in craniofacial pathology.

## Supporting information

S1 Fig CS phenotypes and diagnosis by craniomorphometry. The upper left panel depicts a normal coronal suture with extensive interdigitation. The middle left panel depicts the coronal suture within a DOS rabbit. Note the cluster of bony bridges mid-suture. The lower left panel depicts the fused suture of an EOS rabbit at 10 of age. The upper right panel presents a radiograph showing the placement of silver amalgam markers at suture junctions. These markers are used to measure differences in growth between day 10 and day 25 of age, providing the basis for distinguishing between ICN and EOS rabbits. The lower right panel graphs growth measurements across the coronal suture over time for ICN rabbits, unilateral and bilateral DOS rabbits, and unilateral and bilateral EOS rabbits. Significant differences in growth are observed among the respective groups.
(TIF)

S2 Fig Prioritization of candidate genes for sequencing by Phenolyzer. Candidate genes identified on chromosomes 14 and 19 were prioritized using focused disease/phenotype terms including: bone, cartilage, craniosynostosis, and craniofacial abnormalities.
(TIF)

S1 Table Tagged SNPs used as markers for linkage analysis. The characteristics of the markers used for linkage analysis as displayed in Fig 2 are summarized. CHR = chromosome, POS = position, Case/Control = the minor allele frequency within the respective case and control populations, X^2^ = chi square, ObsHET = observed heterozygosity, PredHET = predicted heterozygosity, HWpval = the Hardy-Weinberg p value, and MAF = minor allele frequency.
(DOCX)

S2 Table Candidate genes located within a single linkage block on chromosome 19. Coding elements located within the region spanning from 34.4 Mb to 34.8 Mb on rabbit chromosome 19 were identified in Ensembl using OryCun2.0 release 91. Gene symbols, genomic coordinates within the OryCun2.0 assembly, and corresponding protein functions as defined in UniProt are reported.
(DOCX)
