# Supplementary figures and images for: Genetic associations and phenotypic heterogeneity in the craniosynostotic rabbit
Source: PLoS One. 2018 Sep 20;13(9):e0204086. doi: 10.1371/journal.pone.0204086 (PMC6147457; doi:10.1371/journal.pone.0204086)

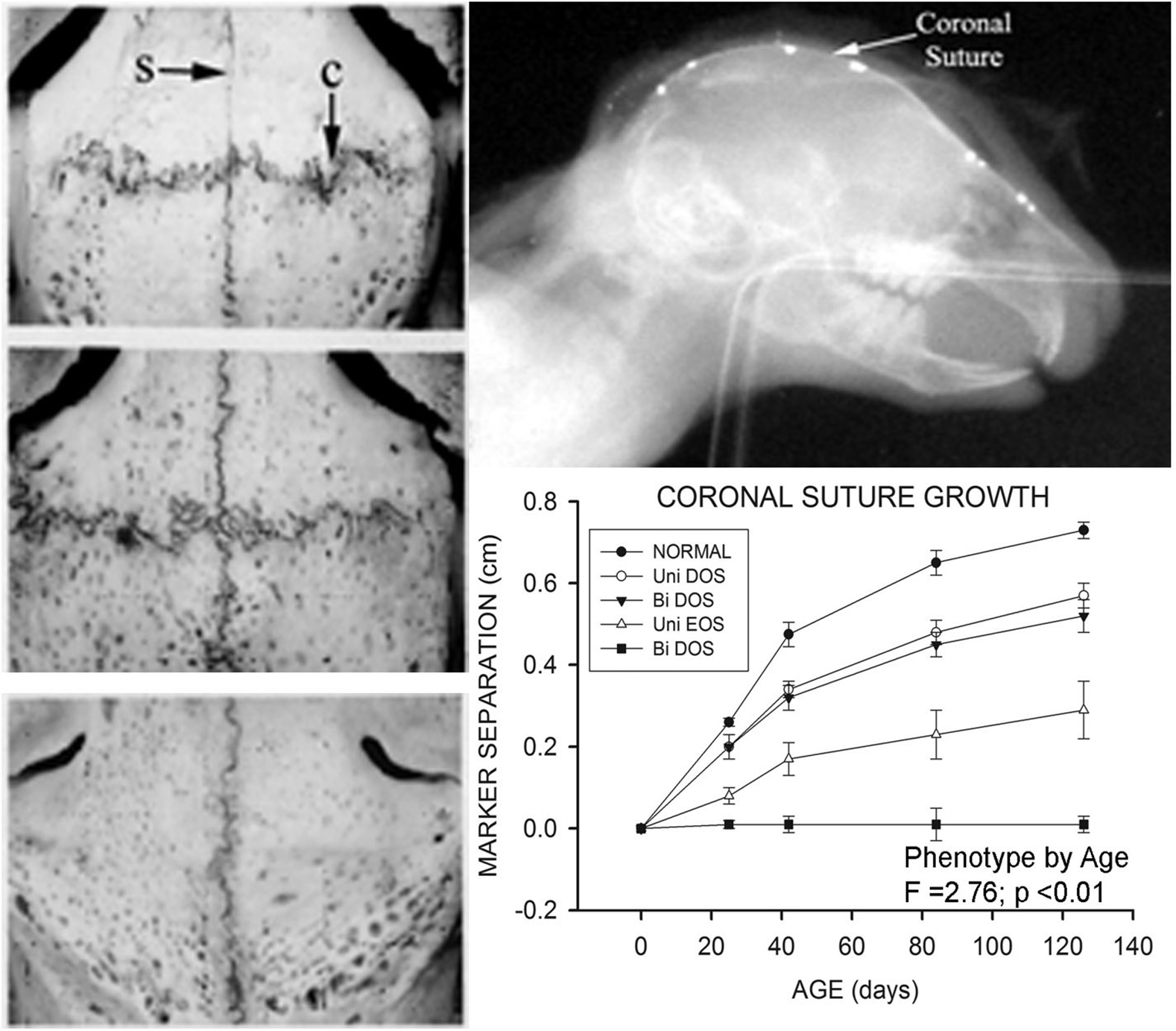

Supplement: S1 Fig — The upper left panel depicts a normal coronal suture with extensive interdigitation. The middle left panel depicts the coronal suture within a DOS rabbit. Note the cluster of bony bridges mid-suture. The lower left panel depicts the fused suture of an EOS rabbit at 10 of age. The upper right panel presents a radiograph showing the placement of silver amalgam markers at suture junctions. These markers are used to measure differences in growth between day 10 and day 25 of age, providing the basis for distinguishing between ICN and EOS rabbits. The lower right panel graphs growth measurements across the coronal suture over time for ICN rabbits, unilateral and bilateral DOS rabbits, and unilateral and bilateral EOS rabbits. Significant differences in growth are observed among the respective groups. (TIF) [file pone.0204086.s001.tif]

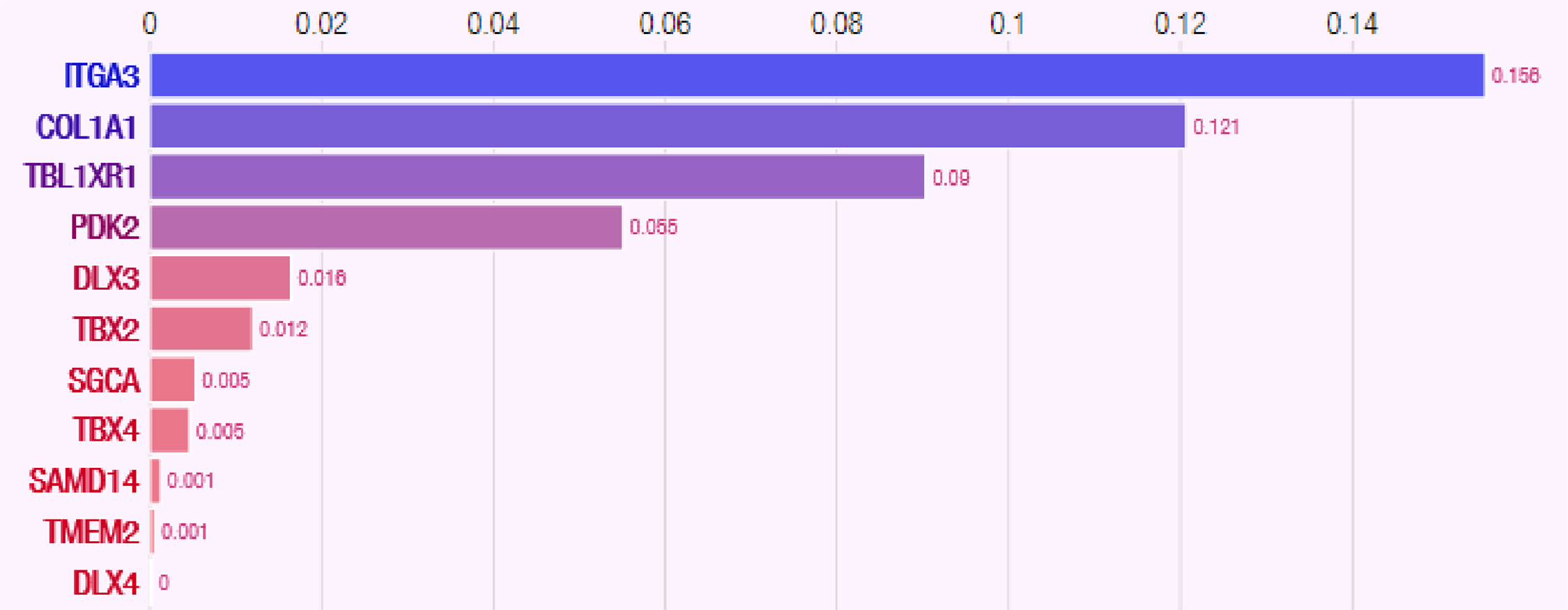

Supplement: S2 Fig — Candidate genes identified on chromosomes 14 and 19 were prioritized using focused disease/phenotype terms including: bone, cartilage, craniosynostosis, and craniofacial abnormalities. (TIF) [file pone.0204086.s002.tif]
