# Supplementary material for: Genetic associations and phenotypic heterogeneity in the craniosynostotic rabbit
Source: PLoS One. 2018 Sep 20;13(9):e0204086. doi: 10.1371/journal.pone.0204086 (PMC6147457; doi:10.1371/journal.pone.0204086)
Supplement: S1 Table — The characteristics of the markers used for linkage analysis as displayed in Fig 2 are summarized. CHR = chromosome, POS = position, Case/Control = the minor allele frequency within the respective case and control populations, X2 = chi square, ObsHET = observed heterozygosity, PredHET = predicted heterozygosity, HWpval = the Hardy-Weinberg p value, and MAF = minor allele frequency. (DOCX) [file pone.0204086.s003.docx]

**S1 Table. Tagged SNPs used as markers for linkage analysis.**

| **CHR** | **marker** | **POS** | **Case,Control** | **Χ^2^** | **ObsHET** | **PredHET** | **HWpval** | **MAF** |
| --- | --- | --- | --- | --- | --- | --- | --- | --- |
| 2 | 1 | 6388292 | 0.750, 0.238 | 22.5 | 0.395 | 0.5 | 0.249 | 0.5 |
|  | 2 | 6393336 | 0.773, 0.250 | 22.9 | 0.286 | 0.499 | 0.009 | 0.476 |
|  | 3 | 6413213 | 0.848, 0.400 | 18.6 | 0.302 | 0.461 | 0.044 | 0.36 |
|  | 4 | 6468739 | 0.795, 0.341 | 18.5 | 0.5 | 0.491 | 1 | 0.432 |
|  | 5 | 6565957 | 0.750, 0.286 | 18.6 | 0.442 | 0.499 | 0.600 | 0.477 |
|  | 6 | 6570299 | 0.792, 0.286 | 23.2 | 0.4 | 0.494 | 0.292 | 0.444 |
|  | 7 | 6588362 | 0.750, 0.333 | 15.1 | 0.395 | 0.496 | 0.27 | 0.453 |
|  | 8 | 6590395 | 0.667, 0.182 | 21.9 | 0.304 | 0.491 | 0.017 | 0.435 |
|  | 9 | 6605091 | 0.021, 0.000 | 0.9 | 0.022 | 0.022 | 1 | 0.011 |
|  | 10 | 6615936 | 0.750, 0.250 | 22 | 0.364 | 0.5 | 0.11 | 0.5 |
|  | 11 | 6622820 | 0.771, 0.250 | 24.9 | 0.435 | 0.499 | 0.512 | 0.478 |
|  | 12 | 6641309 | 0.857, 0.182 | 39.2 | 0.326 | 0.5 | 0.037 | 0.488 |
| 19 | 1 | 37533454 | 1.000, 0.417 | 19.8 | 0.417 | 0.413 | 1 | 0.292 |
|  | 2 | 37562557 | 1.000, 0.458 | 16.6 | 0.304 | 0.405 | 0.415 | 0.283 |
|  | 3 | 37618286 | 1.000, 0.458 | 17.8 | 0.375 | 0.395 | 1 | 0.271 |
|  | 4 | 37654122 | 1.000, 0.542 | 14.3 | 0.292 | 0.353 | 0.659 | 0.229 |
|  | 5 | 37716075 | 1.000, 0.850 | 3.6 | 0.048 | 0.133 | 0.146 | 0.071 |
|  | 6 | 37824932 | 1.000, 0.375 | 21.8 | 0.292 | 0.43 | 0.223 | 0.312 |

**S1 Table. Tagged SNPs used as markers for linkage analysis.** The characteristics of the markers used for linkage analysis as displayed in Fig 2 are summarized. CHR = chromosome, POS = position, Case/Control = the minor allele frequency within the respective case and control populations, Χ^2^ = chi square, ObsHET = observed heterozygosity, PredHET = predicted heterozygosity, HWpval = the Hardy-Weinberg p value, and MAF = minor allele frequency.
