# Supplementary material for: Genetic associations and phenotypic heterogeneity in the craniosynostotic rabbit
Source: PLoS One. 2018 Sep 20;13(9):e0204086. doi: 10.1371/journal.pone.0204086 (PMC6147457; doi:10.1371/journal.pone.0204086)
Supplement: S2 Table — Coding elements located within the region spanning from 34.4 Mb to 34.8 Mb on rabbit chromosome 19 were identified in Ensembl using OryCun2.0 release 91. Gene symbols, genomic coordinates within the OryCun2.0 assembly, and corresponding protein functions as defined in UniProt are reported. (DOCX) [file pone.0204086.s004.docx]

**S2 Table. Candidate genes located within a single linkage block on chromosome 19.**

| **Gene** | **Position** | **Function** |
| --- | --- | --- |
| TMEM2  COL1A1  SGCA  SAMD14  PDK2  ITGA3  DLX3  DLX4  TAC4 | 37,422,398-37,423,124  37,474,894-37,582,234  37,497,298-37,508,002  37,536,109-37,550,873  37,553,073-37,564,679  37,568,632-37,598,932  37,650,520-37,653,752  37,670,259-37,675,009  37,783,651-37,793,713 | hyaluronidase  ECM component  sarcoglycan alpha  sterile alpha motif domain-containing protein  protein kinase  ECM-receptor interaction  transcription factor  transcription factor  hemokinin |

**S2 Table. Candidate genes located within a single linkage block on chromosome 19.** Coding elements located within the region spanning from 34.4 Mb to 34.8 Mb on rabbit chromosome 19 were identified in Ensembl using OryCun2.0 release 91. Gene symbols, genomic coordinates within the OryCun2.0 assembly, and corresponding protein functions as defined in UniProt are reported.
